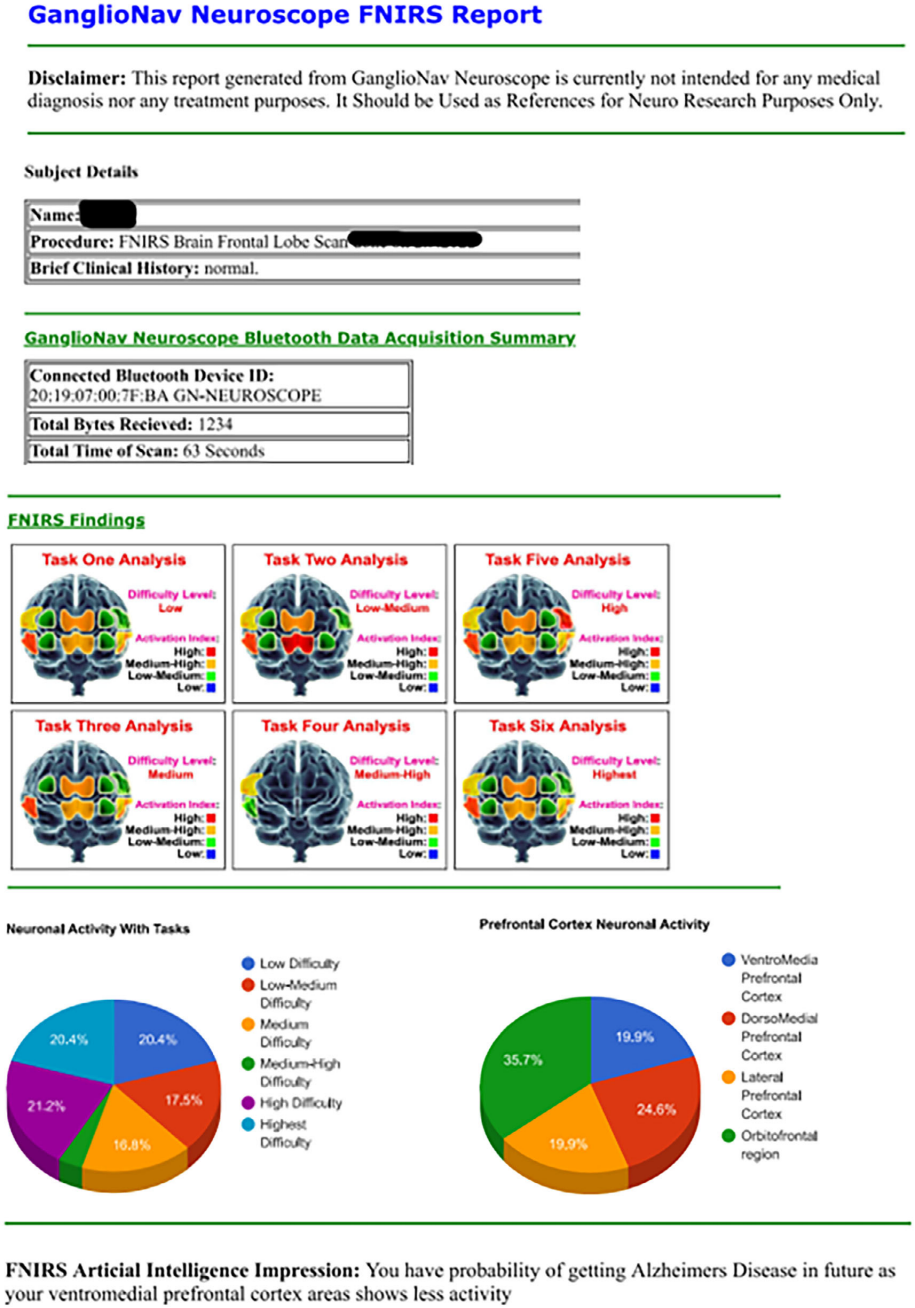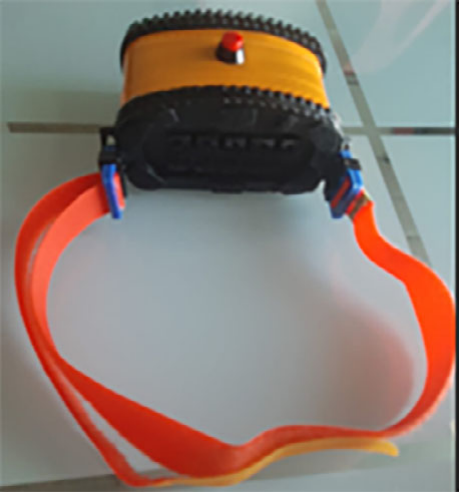# GanglioNav Neuroscope: A Novel Functional Near Infrared Spectroscopy (fNIRS) System for Monitoring Cognitive Function in Healthy Adults

**DOI:** 10.1002/alz70858_100405

**Published:** 2025-12-25

**Authors:** Vinu Sherimon, Sherimon P.C., Rahul V. Nair

**Affiliations:** ^1^ University of Technology and Applied Sciences, Muscat, Muscat, Oman; ^2^ Arab Open University, Muscat, Oman; ^3^ Aster Al Raffah Hospital, Muscat, Muscat, Oman

## Abstract

**Background:**

fNIRS is an advancing neuroimaging modality that provides a distinct insight into human brain activity. It utilizes the unique absorption characteristics of near‐infrared light (700‐900 mm) by oxygenated (HbO2) and deoxygenated (HHb) hemoglobin in the brain. Other tissues exhibit partial transparency throughout this wavelength range, but light intensity changes at 760 nm and 850 nm can precisely quantify hemodynamic variations. These variations are further analyzed as indications of cerebral activity using the Beer‐Lambert rule. This research presents the GanglioNav Neuroscope, an innovative fNIRS gadget for monitoring prefrontal brain activity.

**Method:**

The study included 20 participants (mean age = 24.5, SD=4.2). The device consists of ten sensors and five emitter sets. The forehead‐mounted device measures the variations in HbO2 and HHb concentrations. The device compares light absorption at 760 nm and 850 nm near‐infrared light. Each participant underwent six cognitive tasks to assess prefrontal brain function.

*N‐back* task observed an uninterrupted sequence of letters and identify whether the present letter matched the one shown “N” trials back, the *Stroop* task involved identifying the ink color from a list of color words shown in incorrect ink colors, the *Go/No‐Go* task checked response to a sequence of letters by pressing a button for every letter except for a specified target letter, the *lowa Gambling* task simulates real‐life decision making under uncertainty, the *Trail Making* test to connect a series of numbers and letters in an alternating pattern and *Verbal Fluency* task, to produce as many words as possible within a time frame. An AI application was employed to analyze the fNIRS data. Changes in HbO2 and HHb concentrations were analyzed to assess the hemodynamic response in four key regions of the prefrontal cortex.

**Result:**

The fNIRS data obtained throughout the six cognitive tests demonstrated unique activation patterns across various prefrontal areas. AI‐driven analysis of the fNIRS data yielded personalized cognitive evaluation ratings for each participant. These scores indicated the overall efficiency and efficacy of prefrontal brain function across several cognitive activities.

**Conclusion:**

This research highlights the potential and promise of utilizing fNIRS to evaluate prefrontal brain activity.